# Facial Foramen Diagnostic and Surgical Role as Reference Points in Asymmetries—Cone-Beam Computed Tomography Preliminary Study

**DOI:** 10.3390/jcm14020463

**Published:** 2025-01-13

**Authors:** Kamil Nelke, Maciej Janeczek, Agata Małyszek, Marceli Łukaszewski, Marta Frydrych, Michał Kulus, Paweł Dąbrowski, Klaudiusz Łuczak, Wojciech Pawlak, Grzegorz Gogolewski, Maciej Dobrzyński

**Affiliations:** 1 Privat Practice of Maxillo-Facial Surgery and Maxillo-Facial Surgery Ward, EMC Hospital, Pilczycka 144, 54-144 Wroclaw, Poland; klaudiusz.luczak@gmail.com (K.Ł.); wojciech.pawlak.mfs@gmail.com (W.P.); 2 Academy of Applied Sciences, Health Department, Academy of Silesius in Walbrzych, Zamkowa 4, 58-300 Walbrzych, Poland; 3 Department of Biostructure and Animal Physiology, Wrocław University of Environmental and Life Sciences, Kożuchowska 1, 51-631 Wroclaw, Poland; maciej.janeczek@upwr.edu.pl (M.J.); agata.malyszek@upwr.edu.pl (A.M.); 4 Department of Anaesthesiology and Intensive Care, Sokołowski Hospital, Sokołowskiego 4, 58-309 Walbrzych, Poland; marceliluk@gmail.com (M.Ł.); frydrych.marta.k@gmail.com (M.F.); 5 Division of Ultrastructural Research, Wroclaw Medical University, 50-367 Wroclaw, Poland; michal.kulus@umw.edu.pl; 6 Division of Anatomy, Department of Human Morphology and Embryology, Wroclaw Medical University, 50-367 Wroclaw, Poland; pawel.dabrowski@umw.edu.pl; 7 Department of Emergency Medicine, Wroclaw Medical University, Borowska 213, 50-556 Wrocław, Poland; grzegorz.gogolewski@umw.edu.pl; 8 Department of Pediatric Dentistry and Preclinical Dentistry, Medical University in Wroclaw, Krakowska 26, 50-425 Wrocław, Poland; maciej.dobrzynski@umw.edu.pl

**Keywords:** supraorbital foramen, infraorbital foramen, mental foramen, mandibular asymmetry, condylar hyperplasia

## Abstract

**Introduction:** Facial asymmetry can be attributed to a multitude of underlying causes. Multiple reference points can be utilized for guidance in surgery planning. The scope of mandibular overgrowth and asymmetry should always be measured on CBCT radiographs (cone-beam computed tomography). The assessment of the mental foramen, and the supra and infraorbital foramina is crucial in surgical procedures. Their potential as reference points for predicting specific conditions has never been studied before. The authors explored if the mentioned foramina can be used for diagnostic purposes to distinguish the type of asymmetry or perhaps could improve any surgery planning in those skeletal asymmetry cases. **Material and methods:** Evaluation of 30 CBCT radiographs in the present preliminary study based on three study groups consisting of patients with normal skeletal features without any skeletal malocclusion (*n* = 10), and those compared with hemimandibular elongation (HE = 10) and hyperplasia (CH/HH = 10). For the evaluation of asymmetry, fluctuating asymmetry indices were calculated. **Results:** The fluctuating asymmetry indices did not differ between both sexes; however, they were remarkably higher in the CH groups than in HE or control. Some of the indices showing the highest differences show some potential as promising predictors for early detection of CH. **Conclusions:** The condylar hyperplasia shows the highest facial asymmetry among study groups and metric traits. The supraorbital-mental foramina measurement may be used for initial screening for the occurrence of condylar hyperplasia Additional measurements could increase the predictive value of this indicator. Further study on improved samples could confirm the hypothesis of facial foramina displacement influence on jaw osteotomy planning.

## 1. Introduction

**Figure 1:**
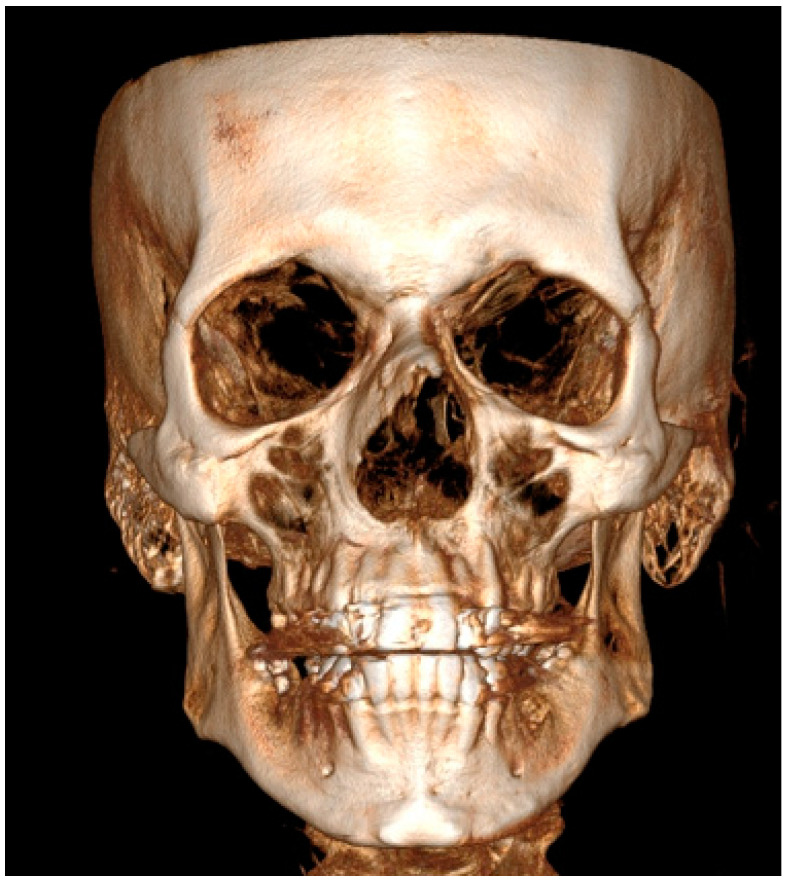
CBCT evaluation with 3D modeling in coronal view of a perfect symmetrical and balanced facial skeleton.

**Figure 2:**
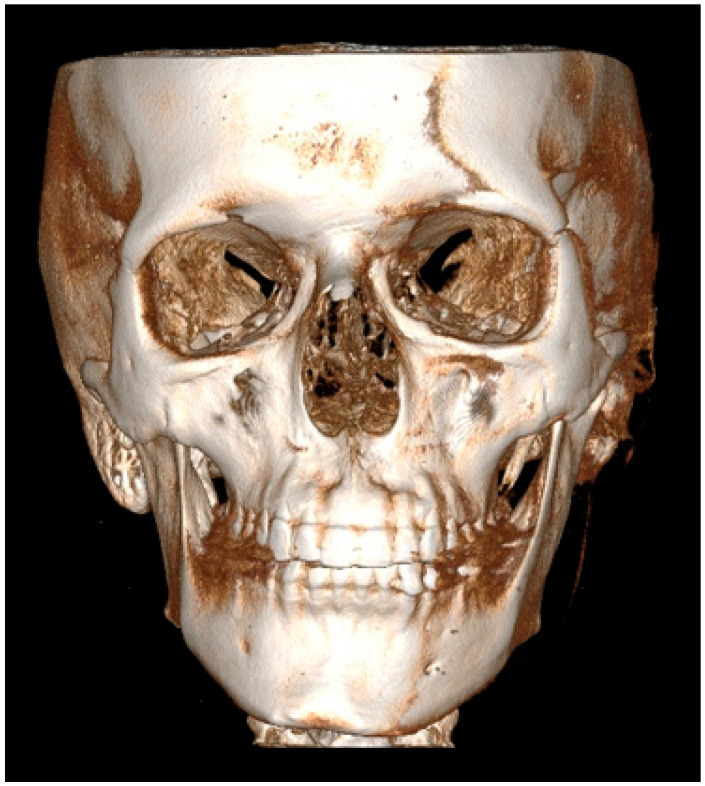
CBCT 3d-evaluation in left-sided mandibular condylar hyperplasia (CH)—an asymmetry noticed in the mandibular left body and chin position followed by deviation in the maxillary cant.

Facial asymmetry can be attributed to a multitude of underlying causes. Both acquired or congenital etiology are possible [1]. It is frequently observed that mandibular asymmetry is associated with either unilateral condylar hyperplasia (CH/HH) or condylar elongation (HE) [1,2]. These pathologies are not that common, however, and when diagnosed they are responsible for some atypical mandibular asymmetry [2]. The aforementioned pathologies (CH/HH) are associated with an atypical, non-neoplastic, mostly unilateral excessive one-sided bone growth of the affected condyle, causing visible mandibular asymmetry in the ramus and body of the mandible. Visible mandibular asymmetry features can be easily compared in radiological CBCT (cone-beam computed tomography) studies (Figure 1 and Figure 2). Over time, CH can grow continuously and can limit itself during time or progress even after puberty, resulting in various forms and degrees of mandibular asymmetry, and even secondary maxillary asymmetry [3,4] (Figure 2). Because of maxillary and mandibular bone asymmetry, it is possible to undertake a detailed examination of several clinical and radiological features. The study on supraorbital, infraorbital, and mental foramen characteristics in cases of mandibular asymmetry (CH/HE) may reveal novel diagnostic features or impact surgical planning. The authors would like to reflect that it has been little studied on the mentioned foramina in condylar hyperplasia and elongation cases, and no similar reference was found [5,6].

**Figure 3:**
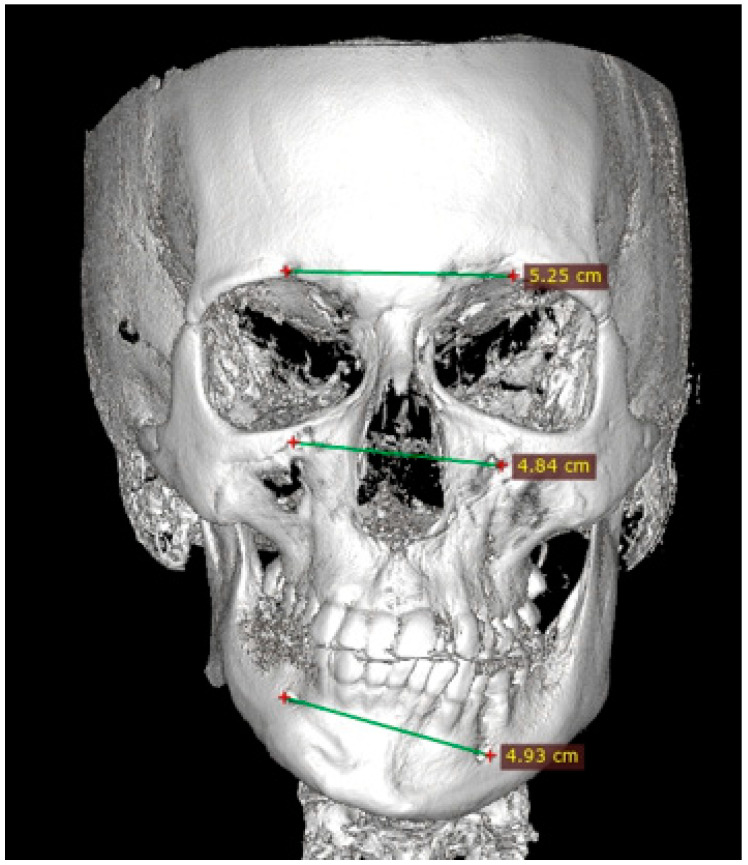
CBCT measurements in maxillo-mandibular asymmetry caused by condylar hyperplasia—from top the SOF, IOF, and MFO measurements in horizontal aspect. Additional horizontal lines indicate the foramina tilting and scope of asymmetry compared to the opposite healthy side and the skeletal midline.

Some anatomical landmarks, including the position of the gonial angle, degree of chin asymmetry, deviations in the shape and position of the lower part of the mandibular body, differences in the ramus height and maxillary bite plane deviation can influence the scope and necessity for any surgical interventions in the facial skeleton to improve facial balance and contour [6,7] (Figure 2 and Figure 3). The position of the key anatomical landmarks, such as facial foramina with key nerve branches of the trigeminal nerve or others can be useful. Furthermore, the scope of asymmetry in various cases can be also compared when other anatomical landmark reference points could be used, for example, the vertical and horizontal distances between the mandibular basis, the mandibular canal, and the occlusal mandibular plane, or others [1,2,3,4,5,6,7,8].

The trigeminal nerve (CNV) is one of the cranial nerves. It is the largest of all cranial nerves and provides the majority of sensory innervation to the face. The CNV is divided into three major branches—the ophthalmic nerve (V1), the maxillary nerve (V2), and the mandibular nerve (V3) [8]. Its main branches arise from the supraorbital (SOF), infraorbital (IOF), and mental foramina (MNO) (or a notch). They are usually distributed in a vertical line (in a coronal view) passing through the middle of the pupil. According to Gupta et al. study, approximately 80% of all the foramina are situated along a single vertical line [9]. A study conducted on cadavers by Hester et al. suggests that there are significant variations in these foramen positions and that they are equidistant from the midline [10]. The author asserts that meticulous planning is essential for any surgical intervention in these areas. This fact is also related to sex differences in the foramen’s localization. As demonstrated by a study conducted by Cutright et al., the aforementioned anatomical and sex-related factors exert a significant influence on the position of the foramen. Consequently, surgical approaches must take these factors into account [11,12].

The mental foramen (MFO) is located at the premolar region on both sides of the mandibular body. A neurovascular bundle consisting of the mental nerve (from the inferior alveolar nerve, IAN) and several small blood vessels arises from this foramen (from the mandibular nerve, V3) [13]. The mental foramen can be subdivided into smaller foraminae which in turn give rise to multiple branches of mental nerve that extend towards the adjacent soft tissues. The study conducted by Nejami et al. indicated that MFO can be easily identified on a routine radiograph. On the other hand, there can be a discrepancy between the observed location and the actual position of the MFO in situ. Consequently, some CBCT evaluations provide a clearer image of the MFO, which is also confirmed by the findings of Sheikhi et al. The distance between the inferior border of the mandible, the skeletal midline and tooth apexes, and MFO provides some valuable information when planning surgical procedures [14,15,16]. The position of MNO is always disrupted in any asymmetry cases, depending on the scope and degree of bone asymmetry. Some authors also used the dimensions between mental foramen, mandibular foramen, and gonial angles in vertical lines as valuable measurement points to compare entire mandible symmetry [12,13,14,15,16,17]. The scope of three-dimensional bone overgrowth in UCH might lead to MFO displacement, similar to a lowe-setted mandibular canal typical for condylar-related asymmetry.

The infraorbital foramen (IOF) is situated beneath the inferior orbital rim, approximately 10 mm below. It transmits the infraorbital artery and vein, and the infraorbital nerve, a branch of the maxillary nerve (V2). The nerve’s origin is located at the inferior part of the orbital floor, extending towards an infraorbital canal and then subsequently towards the infraorbital groove [17,18]. Aziz et al. cadaver study revealed no statistically significant differences between the left and right sides or between the studied sexes. Additionally, the authors noted that the maxillary premolar tooth was most frequently situated in the same vertical plane as the infraorbital foramen, while it is also worth pointing out any anatomical variations of the infraorbital foramen. Moreover, the authors documented cases with multiple ipsilateral accessory foramina in 15% of the cadavers [19,20]. Quite often, IOF asymmetry in mandibular asymmetry is an additional feature, related to the secondary maxillary tilting and bite plane deviation in time while asymmetrical mandibular growth is present. This aspect was also studied in comparison to other anatomical landmarks, like palatal length and width, distances between anterior and posterior nasal spines or mandibular distances between gonial angles, and the scope of the mandibular angle variances [18,19,20,21].

The supraorbital foramen (SOF) is located at the superior rim of both orbital sockets. The supraorbital nerve arises from either a foramen or a notch. Woo et al. study underline that a CBCT-3D study grants a more precise view in the evaluation of SOF anatomy and variations. On the other hand, Haładaj et al. study indicates that SOF and IOF have different nerve fiber patterns [21,22,23,24]. Since these foramina were already studied across many aspects, the authors tried to improve their usage and highlight if they can be also used for something else, in this case as a reference for asymmetry diagnostics or as topographical landmarks while planning some scope of corrective and bone surgeries in the facial area.

The authors hypothesize that the three-dimensional differences between foramina localization can have a different vertical, horizontal, and transverse dimension relation between the mandibular basis, the mandibular plane, and the tooth apices might influence the surgery osteotomy line placement. Furthermore, the authors hypothesize that the scope of three-dimensional mandibular bone overgrowth in UCH might lead not only to a shift or a malposition of the mental foramen, but also influence the vertical and horizontal osteotomy lines near its position. This hypothesis requires more studies in the future, on a bigger group and an improved study sample for comparison.

The objective of the present preliminary study was to delineate differences in measurements between SOF/IOF/MFO points and their asymmetry indices across CH, HE, and control groups and their possible usage as surgery-guided reference points or a diagnostic factor for distinguishing the asymmetry type, as a new and alternative method of diagnostics. Subsequently, the prognostic value of asymmetry indices in predicting CH was assessed. 

## 2. Material and Methods

### 2.1. Study Design

A retrospective preliminary study was conducted based on CBCT 3D scans of the facial skeleton of 30 participants. All participants enrolled in the study consented to the anonymous study. The radiological data obtained from CBCT (field of view: 20 × 20 cm) was used to divide all participants into three study groups. Group N0 (*n* = 10) healthy participants, without any skeletal or mandibular asymmetries (M/F-ratio 1:1); group N1(*n* = 10)- study group consisted of ten UCH/CH patients (M/F-ratio: 1:1) and group N2(*n* = 10)- study group consisted of ten patients with mandibular asymmetry related with HE (M/F-ratio 1:1). The present research is a retrospective study on CBCT radiographs from the authors’ own clinical practice database. The initial phase of the study was to prove if the discrepancies between studied foramina should be studied in the future on a larger sample. The CBCT were studied in adults >18 years of age. These patients had their CBCT performed for various reasons, such as orthognathic surgery, condylar hyperplasia, and evaluation of bone/mandible asymmetry. Patient study groups were identified based on other authors’ studies and databases in the aspect of skeletal and mandibular asymmetries [25,26,27].

Inclusion criteria for the study are CBCT only from the authors’ database; patients treated, diagnosed, or operated on with CBCT 20 × 20 full scope of images; and no relevant medical history concerning any other pathologies in the craniofacial skeleton or its treatment. Exclusion criteria are CBCT from outside of the authors’ patient database; missing radiological/clinical data; CBCT after any possible surgery (such as Lefort I, orthognathic surgery, cystectomy, trauma/fracture cases, or others); and patients not related with the scope of craniofacial skeleton evaluation.

The research respected the ethical principles of the Helsinki Declaration and the CBCT guidelines. For this research to begin, ethical approval was first signed by the Bioethics Committee nr 2-4-BNR-2022 (12 October 2022).

### 2.2. Asymmetry Features

Mandibular asymmetry related to CH(HH) and HE cases has its typical features. While CH is related to an abnormal, excessive prolonged growth in the condylar head, far beyond normal growth time causing skeletal/mandibular one-sided asymmetry in vertical vector, chin shift beyond the facial midline, visible three-dimensional one-sided mandibular overgrowth with typical chin deviation towards the healthy side, occurrence of open bite and bite plane deviation. A bone scintigraphy/SPECT (single-photon emission computed tomography) and a study on bone proportions in CT can indicate unilateral condylar hyperplasia. On the other hand, HE is not related to the abnormal excessive growth in the condylar head, without any morphological, structural, or other changes in the condylar head, with the growth in horizontal vector, causing enlarged ramus and can be found bilaterally in the mandible [1,2,3,4,5,6,7]. There are many anatomical and radiological landmarks and key points that can be used to distinguish the CH/HE from other asymmetry cases, however, the authors would want to analyze if the described foramina in this study might have some diagnostics value, especially reference points that had not been studied before.

### 2.3. Cone Beam Computed Tomography Characteristics

A total of 30 CBCT/LDCT scans were evaluated. All of them were enrolled from the authors’ database, studied, and measured in the RadiAnt Dicom Viewer Software version 2020.2.1 (Medixant, Poznań, Poland). CBCT of the patients included in the study was at a maximum size of 20 × 20 cm. Gathered DICOM images helped to establish a 3D-CBCT visualization of each participant’s facial skeleton anatomy. A skull with perfect symmetry is presented in Figure 1. Each radiological datum was closely evaluated based on the position of the supraorbital, infraorbital and mental foramen. Gathered data and variables have been categorized and put into each specific patient’s archive. Information on the facial skeleton was studied and evaluated.

Radiological data manufacturing included most cases being investigated and diagnosed with CBCT 20 × 20 FOV (field of view) imaging protocol based on RayScan S 5471.3 mGy (RayCompany Ltd., Samsung 1-ro, Hwaseong-si, Gyeonggi-do, Republic of Korea). Patients were positioned by the CBCT device according to the Natural Head Position (NHP) guidelines, while maintaining proper horizontal and Frankfurt orientation lines [8,9,10,11,12,25,26,27].

### 2.4. Methods—Radiological Measurements

**Figure 4:**
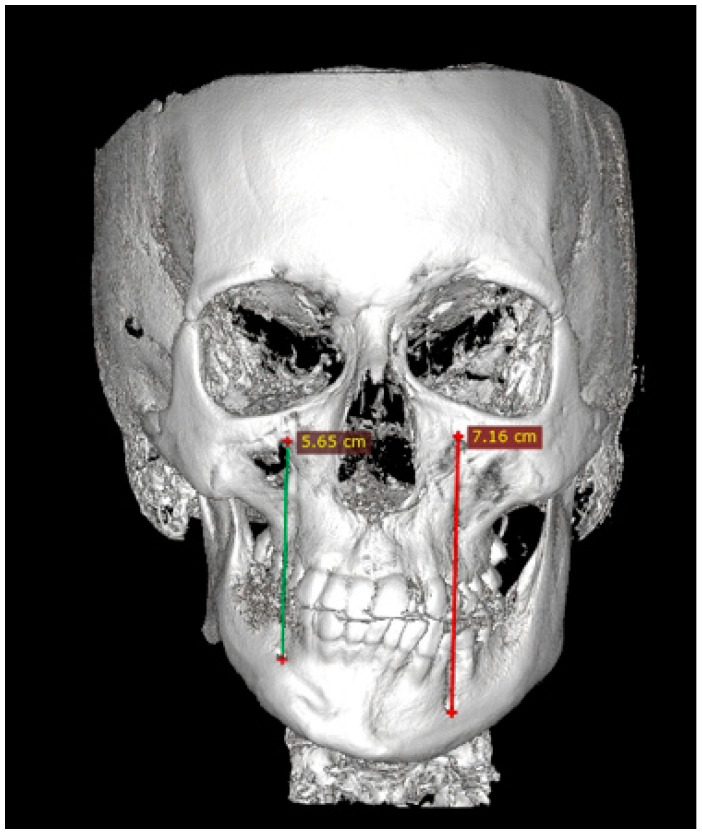
CBCT measurements in maxillo-mandibular asymmetry caused by condylar hyperplasia—the IOF-MFO measurements in vertical aspect.

**Figure 5:**
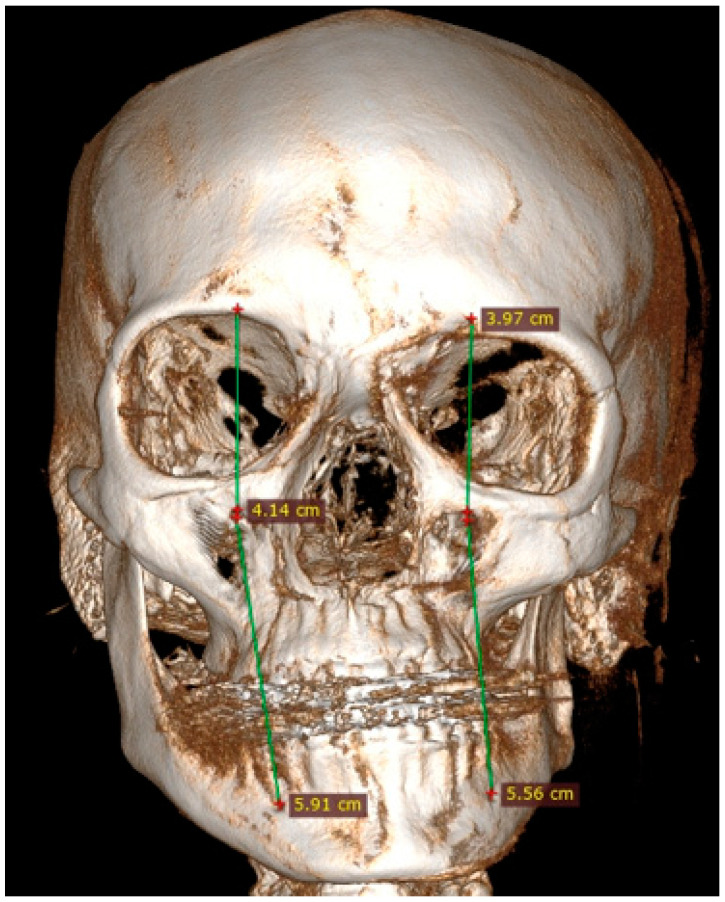
CBCT measurements in maxillo-mandibular asymmetry caused by right-sided condylar hyperplasia—the measurements in vertical aspect: SOF-IOF and IOF-MFO. Vertical lines are useful to indicate the disturbances in symmetry.

**Figure 6:**
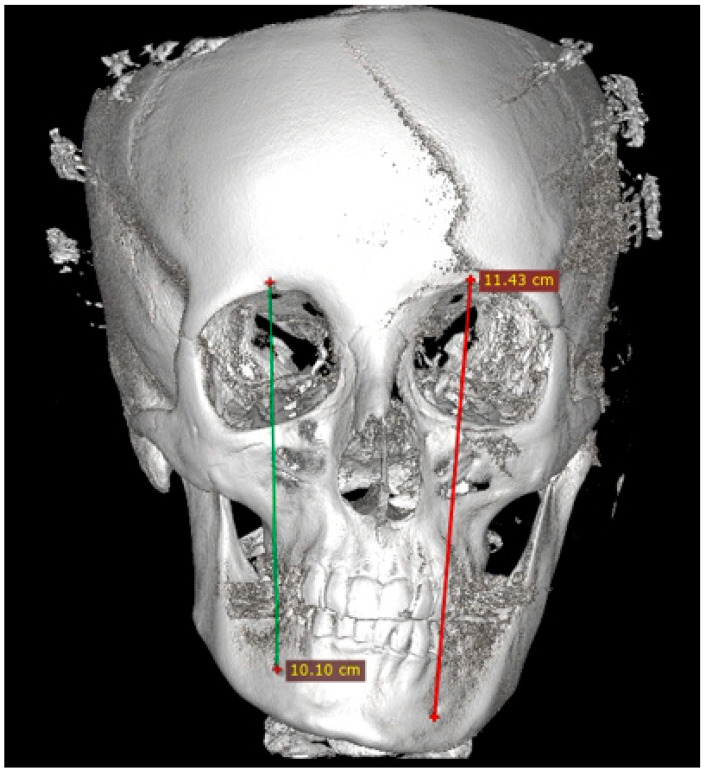
CBCT measurements in maxillo-mandibular asymmetry caused by a right-sided condylar hyperplasia—the measurements in vertical aspect: SOF-MFO. A notable asymmetry and deviations in the foramen position are noticeable.

Before each CBCT radiograph was taken, the patient was situated in a stable and repeatable position where the proper positioning of the head in the CBCT device was ensured with vertical and horizontal reference lines. Each patient was situated according to the natural head position protocol (NHP) [28]. Each CBCT (20 × 20 cm) study was evaluated in a detailed 3D image reconstruction of the facial bones (Figure 1, Figure 2, Figure 3, Figure 4, Figure 5 and Figure 6). Because of a good standardization protocol, it was quite easy to identify and measure the distances between the key anatomical landmarks:

(1) The supraorbital foramen/notch—SOF;
(2) The infraorbital foramen—IOF;
(3) The mental foramen—MFO.

The distances between the foramina were measured. Additional vertical and horizontal lines were used to underline the discrepancies in the foramina position, and scope of asymmetry and to visualize how both vertical and horizontal lines angulation changes within each study asymmetrical group (Figure 3, Figure 4, Figure 5 and Figure 6).

The following measurements and anatomical reference points in horizontal aspect were used:

(1) SOF-SOF distance: between most lateral aspect of the SOF foramen/notch;
(2) IOF-IOF distance: most lateral aspect of the IOF foramen;
(3) MFO-MFO distance: most lateral aspect of the MFO-MFO;

The following measurements in vertical aspect were used:

(1) SOF-IOF right side (R): between most superior SOF and most inferior IOF distance,
(2) SOF-IOF left side (L),
(3) SOF-MFO right side (R): between most superior aspect of SOF and the most inferior aspect of MFO,
(4) SOF-MFO left side (L),
(5) IOF-MFO right side (R): between most superior part of IOF and most inferior part of MFO,
(6) IOF-MFO left side (L).

### 2.5. Statistical Analysis

Given the lack of normal distribution of the majority of evaluated traits, non-parametric tests were employed. For comparisons between males and females, the Mann–Whitney U test was utilized, while the Kruskal–Wallis test was used for comparisons between CH/HE/control groups, with an appropriate post-hoc test employed for the evaluation of differences between distinct group pairs. The statistical significance threshold was set to the *p*-value of 0.05.

To assess asymmetry, the fluctuating asymmetry indices described by Palmer [ref] were employed, specifically FA1 and FA2. FA1 is the absolute value of the difference between the measurements on the left and right sides. FA2 corrects for individual variability in the population and is given by the equation: $\frac{\left|R-L\right|}{(R+L)/2}$, where *R* denotes the measurement on the right side, while *L* represents the measurement on the left.

To evaluate the potential predictive value, receiver operating characteristic (ROC) curves were constructed. The quality of the various predictors was evaluated based on the area under the curve (AUC), sensitivity, and specificity as well as positive and negative predictive value (PPV, NPV) indices. ROC curves illustrate the manner in which the specificity and sensitivity fluctuate when the value designated as the threshold for classification is increased or decreased.

Statistical analysis was performed with Jamovi version 2.5 (Jonathon Love, Damian Dropmann, and Ravi Selker, JASP project, Amsterdam, Netherlands) and packages snowcluster and psychoPDA supported by R environment [29,30,31].

## 3. Results

### 3.1. Differences Between Sexes

The results are presented in Table 1. Statistically significant differences were identified in infraorbital-mental foramen on the right side (*p* = 0.05), supraorbital-mental foramen on the right side (*p* = 0.012), and distances infraorbital -infraorbital foramen (*p* = 0.033) and mental-mental foramen (*p* = 0.003). It is noteworthy that no significant differences were observed in the FA1 or FA2 indices.

### 3.2. Fluctuating Asymmetry Indices Differ in CH/HE/Control Groups

**Figure 7:**
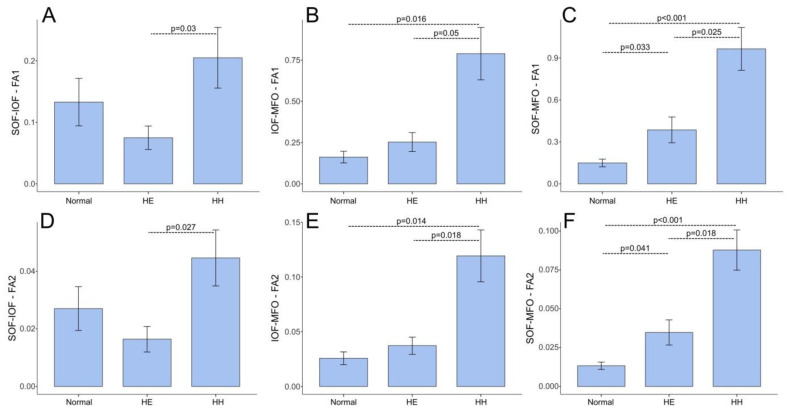
The differences in FA1 (**A**–**C**) and FA2 (**D**–**F**) indices between the condylar hyperplasia CH/HH, HE, and control groups. The *p*-values for the post-hoc test group pair comparisons are shown above the dashed lines. Notably, the infraorbital-mental foramen and supraorbital-mental foramen FA1/2 indices in the condylar hyperplasia (CH) group are significantly higher than those in the hemimamandibular elongation cases (HE) and control groups.

Table 2 presents the results of the Kruskal–Wallis test for differences in distinct measurements between the CH, HE, and control groups. Figure 7 illustrates the selected bar plots that demonstrate the most significant differences.

Notably, the CH group exhibited considerably higher FA1 and FA2 indices than the HE and control groups, which (besides the SOF-MFO distance) demonstrated similar values. Consequently, in the pursuit of predictive values for the evaluated measurements and indices, the CH group was identified as the primary focus of the prediction (*p* < 0.05).

### 3.3. Evaluation of the Predictive Value of FA1/2 Indices

**Figure 8:**
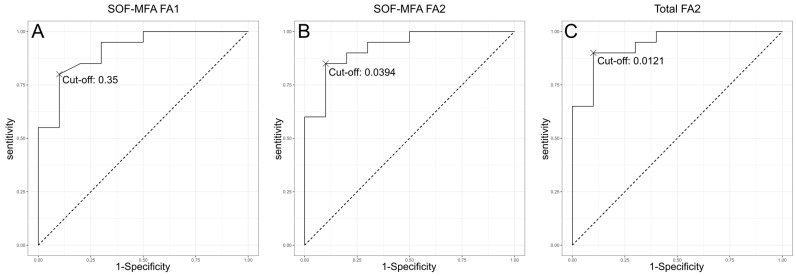
ROC curves for the most promising predictors. (**A**,**B**) is the corelation between supraorbital foramen and mental foramen in FA1 group and FA2, while(**C**) it’s the total value of cut off.

The predictive values of the FA1 and FA2 indices were evaluated using receiver operating characteristic (ROC) curves. Table 3 presents the sensitivity, specificity, positive predictive value, negative predictive value, and area under the curve for the most optimal cut-offs. Figure 8 illustrates the receiver operating characteristic (ROC) plots for the most optimal predictors identified in the current study. In addition to the evaluation of the FA1 and FA2 indices for distinct pairs of measurements, the sum of all FA indices was also assessed.

The greatest predictive value was demonstrated by SOF-MFO and the sum of all FA2 indices, with an AUC exceeding 0.9. The sum of all FA2 indices achieved 81.8% PPV and 94.7% NPV. The mental foramen was associated with a more asymmetrical position (*p* < 0.05), compared to the supraorbital foramen (*p* > 0.05) in condylar hyperplasia and elongation cases.

### 3.4. Role of CBCT on Foramina Study

The present study emphasizes that cone-beam computed tomography is a very good and accurate diagnostic tool. It should always be used in any cases of skeletal asymmetries, especially those scheduled for any surgical intervention. Presenting deviations in both vertical and horizontal measurements on studied foramina, indicated that any condylar hyperplasia asymmetry cases can be easily diagnosed and etiology confirmed based on the results. The differences in the location in both horizontal and vertical planes of all studied foramina are directly correlated with the degree of skeletal changes within their proximity. From a clinical point of view, it is unlikely to have two similar, identical asymmetries or bone disturbances, therefore some anatomical reference points usage can influence future outcomes. Furthermore, some foramina might have some sex and anatomical-related variables that might influence on their shape and position, however the additional vertical and horizontal reference line could still present their angulation, tilting, and proportions when evaluated.

### 3.5. Hypothesis for Future Surgical Consideration

The scope of each osteotomy line placement should take into consideration skeletal asymmetry. In cases of three-dimensional bone overgrowth in UCH, similar to severe asymmetries of other kinds, it is important to estimate the location of the mental foramen (MFO). Its position is the most common one to change and be malpositioned in bone three-dimensions. More superficial localization towards the superior aspect of premolars or more anteriorly placed location towards the incisors could possibly influence its damage or palsy in mandibular osteotomy protocols. On the other hand, the tilted maxillary bite plane with coexisting maxillary deviation might not only influence on lower positioned IOF but also its malposition away from the horizontal line drawn between SOF-IOF-MFO. Furthermore, any segmental maxillary osteotomy or modified Ferguson USSO (unilateral sagittal split osteotomy) osteotomy could be safer if such a position of foramina in severe asymmetries were looked into in the future. The mentioned foramina location in asymmetry cases might only be used as indicators for asymmetry types but could also be helpful in the estimation of surgery planning. Their localization, deviation from the facial midline, and overall usage as reference points for 3d-osteotomy guided-models should be evaluated further in improved study samples in future studies.

## 4. Discussion

The present preliminary study is perhaps the first attempt conducted to estimate if any of the reference points in the supraorbital, infraorbital and mental foramen can be used as potential reference points in the planning of surgery in any asymmetry cases in craniofacial surgeries, or perhaps those foramina can be used alone as references to identify the potential asymmetry etiology. Since no similar reports exist in the literature, the authors tried to establish any utilization of the presented protocol. So far, the results are promising, but are greatly related to the scope of asymmetry, bone overgrowth, and the pattern of asymmetry [25,26,27]. The distances between the mentioned foramina as well as vertical and horizontal lines drawn between those points might indicate some shift or rotation of these lines and foramina position compared to the facial or skeletal midline. It hypothesizes that the scope and type of asymmetry influence the foramina position and, therefore, each planning of osteotomy lines in the mandibular and maxillary bones should be carefully evaluated.

During recent years, the mentioned foramina were the topic of many anatomic and cadaveric studies [20,21,22,23,24,25,26,27,28,29,30]. The disturbances in horizontal and vertical discrepancies in foramina placement in the authors’ study might help each clinician evaluate what type of asymmetry can be found, and also improve planning for each osteotomy protocol for asymmetry correction.

Many craniofacial skeleton abnormalities, such as skeletal malocclusion or asymmetry cases often require some surgical approaches, after their careful radiological evaluation. Additional surgical 3D/virtual planning software and guides are often used to improve each surgery aspect. The main goal of each surgery is focused not only on the restoration of good jaw position, improved bite, masticatory function, occlusion, and esthetics, but also on improving facial symmetry and restoring adequate facial contour. Despite each etiology possibly having many etiological factors, each surgery should be focused on the restoration of symmetry and facial oval [32,33]. In cases of the present foramina in asymmetry surgery, their shape, position, and placement within the facial bones influence the scope and degree of bone osteotomies and also reduces their possible nerve palsy. Some authors also performed additional measurements, especially in the zygomatico-frontal suture, the mental protuberances, nasal bones, external acoustic canal, masseter protuberance, or even other points known from basic cephalometric studies. This helps in facial bone symmetry evaluation and preparation for each osteotomy protocol [1,2,3,4,5,15,19,20,21,22].

Investigation performed in the current study revealed an excess of asymmetry in condylar hyperplasia and elongation. While asymmetry in elongation tends to be similar to that of the control group (except for the SOF-MFO distance), CH excels in asymmetry indices, both in FA1 and FA2. It is therefore imperative that this trait should be taken into consideration during the planning of operations for patients with CH. Furthermore, the differences observed in the supraorbital–mental foramen distance are sufficiently pronounced to warrant consideration as a potential predictor of CH [2,9,12,18]. Because such foramina position and correlations are not usually used, the presented measurements could be used as an additional, indirect point, while the most typical ones have already been widely described in the literature [1,2,3,4,5,6,7,25,26,27]. It further hypothesizes that the scope of mandibular bone three-dimensional overgrowth might cause a significant malposition of the mental foramen in the same way as the UCH is characteristic of a low-stated mandibular canal placement, close to the inferior. This fact, however, should be studied in further studies.

It is noteworthy that a relatively straightforward procedure, namely the summation of the FA2 indices for all three measurements, demonstrated the greatest predictive value. The authors also want to point out that each scope of bone asymmetry and overgrowth in time in each individual case greatly impacts the facial foramina in terms of possible disruptions in their position on various vertical and horizonal reference lines (Figure 3, Figure 4, Figure 5 and Figure 6). Since there are few studies that exist to confirm these significant variables, perhaps this preliminary study will persuade authors for further studies. This highlights the multilevel character of the asymmetry caused by condylar hyperplasia. The main factors resulting from facial skeleton asymmetry are related to cases of laterognathia, condylar hyperplasia, and/or elongation, maxillary hyper/hypoplasia or other secondary-related factors causing either mandibular, maxillary or maxillo-mandibular asymmetry cases. Other cases of facial and mandibular asymmetries might include hereditary, genetic, acquired, iatrogenic, or many other factors [34,35]. All of the present bone asymmetries might cause some malposition or deviation in the position of the supraorbital, infraorbital and mental foramen [1,2,3,4,5,6,7,8,9,10,11,12]. This preliminary study showed the scope of asymmetry and its etiology influence on the facial foramina position, however further studies are required, also on selected groups of patients with a different etiology of asymmetry.

In some cases the asymmetry is not found in the mandible, but the mandible deviation is a secondary finding because of the underdeveloped or hypoplastic maxillary bones and sinuses. It is also worth pointing out that an MSH (maxillary sinus hypoplasia) and malar area symmetry can be also correlated with the anatomical variations of the positioning of the infraorbital foramen, especially when some inferior orbital rim anomalies are present [36]. In all of those, a detailed CBCT study improves the diagnostics and planning for any future surgery. Discrepancies in measurements presented herein between the studied foramina highlight that the SOF, IOF, and MNO positions used are greatly related to the scope of asymmetry and bone position. The authors performed this preliminary study on mandibular asymmetries caused only because of various diseases and conditions affecting the mandible alone. A future perspective and conclusion from this study indicates that asymmetries related with maxillary bone deviations, hypoplasia, aplasia, silent-sinus pathologies, or similar have to be studied separately to see their impact on key facial foraminas.

Since current CBCT/3D studies have a large amount of measuring options, the scope of possible studies based on anatomical landmarks is growing. It is worth understanding that a normal asymmetry is something different from the asymmetry caused by condylar hyperplasia, elongation, or even hemifacial microsomia, since the scope of those changes correlates with three-dimensional changes in bone, teeth, and soft tissue structure changes [1,2,3,4,5,6,7,8,9,10]. It is also worth mentioning that the maxillo-mandibular tilting and bone height along with its anterior rotation could hypothetically also influence the foramina position.

Mandibular-related factors, such as mandibular hyperplasia or elongation, are those most commonly causing visible mandibular asymmetry, while maxillary asymmetry is secondary to the present mandibular pathology. Material gathered by the authors focuses on the differences in the position of the supraorbital, infraorbital and mental foramen and their possible usage in surgery planning. At first, it is important to understand that each position of foramina can influence the selection of the most accurate osteotomy protocol to avoid any possible nerve damage or palsy. Secondly, the position of the foramina, notch, or fissure can estimate what degree of bone overgrowth or bone loss is found at the place of further surgery. Lastly, the variances between the horizontal and vertical positions of the present reference points could be used as additional reference points to estimate the scope of bone volume overgrowth, asymmetry, atrophy, or loss for estimating the most accurate surgical approach for each of those cases. The authors emphasize that the supraorbital foramen is most stable in shape and position, while the mental foramen has the most disturbances in both its vertical and horizontal position. Similarly, in studies by Sheikhi et al. and others, the mental foramen had the most changes in its location [15,16,17,25,26,27,28]. The vertical and horizontal lines used by the authors that connect various facial foramina can improve the perception of asymmetry because the position of each reference line can have different positions, angulation, and rotation towards the facial midline and the opposite side (Figure 3, Figure 4, Figure 5 and Figure 6). These lines should be taken under consideration while evaluating the 3D/CBCT projections of NHP-natural head position placement, as presented by Meiyappan et al. [28].

The position of all mentioned foramina was well studied in the world literature based on anthropometric, cadaveric, CT, radiographic and many other studies and evaluations [8,9,10,11,12,13]. The 3D-CBCT presented in this preliminary study points out the scope and degree of not only vertical and horizontal asymmetry influence, but also the bone in-depth positioning of the mentioned foramina; however, it is important to correlate those findings in the future in improved study samples. Most of the studies emphasize that their location is essential for dental surgery, esthetic medicine, determination of facial and sex differences, and is also important in cases of any plastic and corrective facial surgery. This was also mentioned in studies like Al-Juboori et al., Hester et al., Bahşi et al. and others [10,16,20,24,25,26,27]. In cases of condylar hyperplasia and elongation, because of the discrepancies in mandibular body and angle shape, size and angulation, the position of MFO in relation to SOF, IOF, and the midline and both horizontal and vertical lines can be greatly disturbed [1,2,3,4]. These reference lines can also visualize the difference between the present foramina positions (Figure 3, Figure 4, Figure 5 and Figure 6). The distances, angulation, proportions and correlation between each foramen could also be helpful diagnostics for any surgeries in the facial skeleton. From the author’s perspective, facial foramina, sutures, and other anatomical landmarks are quite good reference points. They can be used not only as symmetry points but also as indicators for possible vertical and horizontal bone overgrowths. The disturbances in their location and position could be used in some cases as indicators for surgery, however, their usage can lead to some misdiagnostics and inappropriate bone measurements [12,13,14,15,16,17,18,19]. The authors were unable to correlate the foramina location with sex or the scope of bone overgrowth and scale of asymmetry, because that requires a lot more studies to prove any correlation within certain age groups as well (Table 2 and Table 3).

The differences in the position of each foramen notable in CBCT could be used as an indicator for each surgeon to perform more detailed and comprehensive surgical planning before surgery. The role of CBCT, CT and good diagnostics were already mentioned by the authors [25,26,27]. Anatomical relations between the mentioned foramina have been well studied in the world literature. Despite the usage of various facial skeleton foramina, incisura and other anatomical landmarks usage for nerve blocks, their localization should be also avoided to reduce any possible nerve damage, palsy, or injury during various surgeries that could be performed in the craniofacial skeleton [30,31,32,33,34,35,36,37,38,39,40,41]. The study by Gupta measured 79 dried human skulls in order to asses and estimate the position of the supraorbital, infraorbital, and mental foramina [9]. On the other hand, the Gupta et al. study indicates that the SOF was about 25 mm from the midline and 30 mm medial from the temporal crest, and 2–3 mm superior from the supraorbital rim. Those measurements are quite interesting in surgical planning for osteotomies or fracture treatments. On the other hand, IOF was located about 7 mm below the inferior orbital rim border and about 28.5 mm apart from the facial midline, while MFO was located about 13 mm from the inferior mandibular border while being 25.8 mm apart from the facial midline. Those results by Gupta et al. indicate that the knowledge of the placement of those anatomical foramina in the facial skeleton is quite important [9]. Nevertheless, it is important to understand that some individual patient characteristics and anatomical variations of the foramina should always be taken into consideration. On the other hand, the 3D-CT evaluation of bones enables good identification of bone anomalies, and especially the location of the mandibular canal in asymmetrical mandibular bone, which is confirmed by Yáñez-Vico et al. and Shekhar et al. [42,43]. Similar findings were noticed in the authors’ study, when the angulation between IOF and MFO might be different, and secondly, that those points in CH cases are not situated on a straight vertical line connecting all three studied points. The authors hypothesize that secondary maxillary tilting and asymmetry related to mandibular asymmetry might also cause significant distortions in the mentioned foramina position, which could influence the segmental osteotomy protocols in Lefort I and II [35,36,37,38,39,40,41].

It is important to note that the disturbances in the position and location of the foramina can indicate a facial and skeletal asymmetry [30,31,32,33,34,35,36,37,38,39,40]. Lack of balance and harmony in some cases might require some surgical approaches, therefore the understanding of some anatomical proportions and positions of the foramina is important. In some cases, a typical foramen can be clinically or radiologically visible as a notch or a groove, which just confirms that anatomical variations in them not only correlate with their shape, size, and position, but also their individual characteristics [1,2,3,4,8,9,10,11,12]. On the other hand, the knowledge of the location of the SOF, IOF, and MFO are important key anatomical reference points for local anesthetic administrations and nerve blocks [15,16,17,18,19,20]. A lack of adequate analgesia and sufficient nerve block might lead to patient pain, discomfort and other troublesome features [34]. In the Song et al. study, the distance between the bilateral IOF (54.9 ± 3.4 mm) was greater than that between the bilateral MFO (47.2 ± 5.5 mm)., while in the authors’ study, the MNO foramen position in CH is unpredictable because of the bone three-dimensional overgrowth. On the other hand, it is more possible to estimate the positions of MFO in healthy and HE patients. Song et al. also evaluated the distances between IOF from the nasal ala, the MFO from mouth angles/chelions and their relations. The surgical implications for mental foramina were presented by Fontenele et al., while the infraorbital were studied by Bahşi et al. [40,41]. Those studies indicate that it would be advisable to measure facial foramina in relation to soft tissue or skin reference points such as the mouth angles, lower lips, nasal ala, midline draw by the pupil, or others.

Both radiologic and clinical evaluation between the mentioned foramina is useful. Smith et al. evaluated the position on fourteen embalmed cadavers and concluded that the studied palpable landmarks are important and useful for planning any surgical approaches in patients with missing teeth or fractures of maxillary bones [38]. This led to a conclusion that both clinical and radiological evaluation can be important for estimating the most accurate surgical approach and minimizing any possible nerve palsy or overestimation of bone discrepancies. Similarly, the usage of new virtual models, 3D devices and surgical guided models were also presented as a valuable additional tool to minimize potential complications and improve outcomes [25,26,27].

Each study on facial bone landmarks, such as sutures, foramina, notches, fissures and other anthropometric landmarks is important for surgery planning [1,2,3,4,5,6,7,8,9,10,11,12]. Studies on mental foramina were made by Udhaya et al., based on measurements of 90 dry adult human mandibles from the south Indian population [39]. The authors measured the position of the foramina, shape, orientation, and presence of any accessory foramen and concluded that the mental foramina were mostly located near the root of 2nd mandibular premolar in the midway between the mandibular inferior margin and alveolar margin of the mandible [39]. This can be found in healthy, asymmetry-free patients; however, in CH, the MFO malposition in three diameters on the overgrowth bone in CH might be considered as its indicator for this pathology. On the other hand, infraorbital foramen shift in vertical position was related to the scope of condylar hyperplasia overgrowth, however, those conclusions need more study in the future [38,39,40,41].

What is quite important, and worth remembering, is that other reference points are also used to establish the scope and degree of each bone overgrowth and asymmetry in unilateral condylar hyperplasia. Most significantly, a low-positioned mandibular canal in the overgrowth one-sided mandibular body is its most common characteristic feature where their three-dimensional vertical bone measurements are used to establish its correlation between teeth apexes, mandibular basis and the buccal and lingual bone cortical plates [1,2,3,4,5,6,7,22,23,24,25,26,27,28]. On the other hand, this preliminary sample included only the Polish population, therefore some anatomical and topographical disturbances on presented foramina might be noted. Furthermore, no similar study was found.

CBCT studies on the mentioned foramina are quite common in orthognathic surgery [19,20,21,22,23,24,25,38,39,40,41,42]. Most commonly, the mental foramina have been studied, because of their direct relation with the mandibular osteotomy protocols [40]. Despite the differences in the location of mental foramina in either second or third skeletal class malocclusion, the CBCT grants a good visualization and insights on the foramina position; nevertheless, the position of the foramina in mandibular hyperplasia or elongation cases has not yet been fully evaluated and presented. The authors have already reported on how the scope of asymmetry, bone overgrowth and discrepancies in bone height and shape influence surgery planning [25,26,27]. In the authors’ study, we conclude that if some vertical and horizontal reference lines between the mentioned foramina are marked on each CBCT-3D study, the gathered proportions might be helpful in surgery planning in both osteotomy or camouflage surgery cases. The fluctuating asymmetry indices did not differ between both sexes, however, they were remarkably higher in the CH groups than in HE or control. For more precise anatomical consideration of the study, sex, bone overgrowth and asymmetries need to be addressed in further studies.

On the other hand, IOF in CBCT has already been widely studied and reported [41]. However, so far the variables in the location and position of IOF in asymmetric mandible, facial asymmetry, and secondary maxillary asymmetry caused by hemimandibular hyperplasia or elongation is not described, therefore the current study might underline some interesting clinical and radiological considerations [20,21,22,23,24,25,26,27]. Their usage was also studied in comparison to the position and location of other anatomical landmarks, such as the mandibular canal, mandibular ramus height, width and angulation, as well as the mandibular foramen position, condylar length and maxillary bite plate relation, or even others [40,41,42,43]. From the authors’ perspective, not only mandible asymmetry and hyperplasia can affect IOF, but also maxillary bone and sinus condition itself. The usage of the mentioned foramina can have various relations with many mandibular, maxillary and facial skeleton bones, therefore, they are quite commonly used in many studies [1,2,3,4,5,6,7,8,9,10,11,12].

It is worth noticing that all of the following authors reported variations in size, shape, and position of the foramina studied in their papers [5,6,7,8,9,10,11,12,13,14,18,19,20,21,22,23,24,25,37,38,39,40,41,42]. This might be not a surprise, but the location between different foramina is dependent on many factors; however, no adequate paper describes the variations between those foramina based on mandibular and skeletal asymmetries related to condylar hyperplasia or elongation patients. In the present study, the authors tried to evaluate the mentioned foramina, the distances between them, and their vertical and horizontal relations, and if they might be useful for surgery planning or diagnostics purposes. Since more adequate, sufficient, and more precise diagnostic, radiological, and clinical landmarks are known, the method described by the authors is an alternative approach. Furthermore, when some asymmetry is noted on a CBCT, this might be an additional factor for each surgeon to use more precise surgical planning software in the preoperative settings. Each individual patient’s characteristics should also include the changes in soft tissue contour, shape and facial morphology when any surgery is planned, especially when using a patient’s individual implant solutions or preparing cutting guides.

Each clinician should be aware that skeletal anomalies in the mandible can secondarily affect the maxillary bone shape, size, and sinus volume, as well as the positioning of the infraorbital foramen. From a clinical point of view for general dentists, the main issue should be focused on the adequate injection of local anesthetics in the close proximity of the foramina, which can be displaced and lead to either insufficient anesthetics or damage to the nerve bundle itself. For otolaryngologists, the secondary disturbances might lead to possible nerve damage during FESS/ESS surgery or possible anatomical disturbances in sinus surgeries. Because the scope of bone asymmetry influences not only the foramina, but also the temporomandibular joint structure, possible ear-related symptoms (like clicking, pain), disturbances in facial skeleton symmetry and sinus asymmetry itself, this problem should be highlighted [25,26,27].

Study limitations in the present preliminary study include the following: a small number of patients (despite this UCH/HH, HE cases are not very common); some diagnostic and surgical features whose meaning require further studies; the necessity to conduct more studies on asymmetry cases; the limitation of CBCT because a more detailed presurgical planning software is needed to improve overall surgical aspects; some sex and anatomical related variables; the studied foramina measurements in asymmetry cases caused by CH/HE never having been addressed before from the diagnostic/surgical aspect, which is why their usage in both surgical and diagnostic aspects is mostly indirect; and the lack of any more key anatomical landmarks for comparison. Further improvement on the soft tissue layout investigation and soft tissue contour calibration on each asymmetry bone as preparation for any surgery is necessary in future studies.

## 5. Conclusions

All facial asymmetry cases influence the position of the facial foramina. Hemimandibular hyperplasia has the most impact on the disturbances of the mental foramina. The condylar hyperplasia shows the highest facial asymmetry among study groups and metric traits. The supraorbital-mental foramina measurement may be used for initial screening for the occurrence of condylar hyperplasia, although standardization would require a study on a larger cohort. Summing the asymmetry indices can be useful for screening CH; additional measurements could increase the predictive value of this indicator. Further study on improved samples could confirm the hypothesis of facial foramina displacement’s influence on jaw osteotomy planning.

## Figures and Tables

**Table 1:** Differences of evaluated measurements between males and females. The significance of differences was established with Mann–Whitney U test. Significant *p*-values (*p* ≤ 0.05) are bolded.

	Group	Mean	Median	SD	*p*-Value
SOF-IOF right	Female	4.5473	4.7200	0.4465	0.455
	Male	4.7300	4.7000	0.3205	
SOF-IOF left	Female	4.5373	4.6700	0.4803	0.262
	Male	4.7473	4.7600	0.3077	
SOF-IOF FA1	Female	0.1233	0.1200	0.0847	0.917
	Male	0.1520	0.1000	0.1613	
SOF-IOF FA2	Female	0.0269	0.0259	0.0176	1.000
	Male	0.0319	0.0215	0.0328	
IOF-MFO right	Female	6.1920	6.2200	0.6893	0.050
	Male	6.7920	6.7800	1.1101	
IOF-MFO left	Female	6.4413	6.5200	0.6433	0.787
	Male	6.5080	6.4300	0.8617	
IOF-MFO FA1	Female	0.3867	0.2700	0.4288	0.740
	Male	0.4160	0.2500	0.4130	
IOF-MFO FA2	Female	0.0618	0.0434	0.0687	0.838
	Male	0.0597	0.0368	0.0567	
SOF-MFO right	Female	10.5927	10.5700	0.7354	0.012
	Male	11.3607	11.2500	0.8376	
SOF-MFO left	Female	10.7967	10.8300	0.6103	0.152
	Male	11.1080	11.0000	0.5565	
SOF-MFO FA1	Female	0.4747	0.2500	0.4526	0.693
	Male	0.5260	0.3300	0.5064	
SOF-MFO FA2	Female	0.0445	0.0226	0.0418	0.806
	Male	0.0461	0.0277	0.0434	
IOF-IOF	Female	5.2087	5.2200	0.3662	0.033
	Male	5.5587	5.6100	0.4890	
SOF = SOF	Female	5.1813	5.2000	0.5478	0.003
	Male	5.6933	5.5200	0.5507	
MFO-MFO	Female	4.7807	4.7600	0.2190	0.319
	Male	4.9480	4.8800	0.4803	

Abbreviations The supraorbital foramen/notch—SOF; The infraorbital foramen—IOF; The mental foramen—MFO; SD-standard deviation, *p* < 0.05 significant value.

**Table 2:** Kruskal–Wallis tests results for comparison on distinct measurements in CH/HE/control groups. Significant *p*-values (*p* ≤ 0.05) are bolded. Notably, only results for FA1/2 indices may be considered statistically significant.

	χ^2^	df	*p*
SOF-IOF right	4.614	2	0.100
SOF-IOF left	2.718	2	0.257
SOF-IOF FA1	6.466	2	0.039
SOF-IOF FA2	6.575	2	0.037
IOF-MFO right	0.932	2	0.628
IOF-MFO left	1.521	2	0.468
IOF-MFO FA1	9.549	2	0.008
IOF-MFO FA2	10.655	2	0.005
SOF-MFO right	1.879	2	0.391
SOF-MFO left	0.576	2	0.750
SOF-MFO FA1	17.270	2	<0.001
SOF-MFO FA2	17.809	2	<0.001
IOF-IOF	0.895	2	0.639
SOF = SOF	0.458	2	0.796
MFO-MFO	1.932	2	0.381

Abbreviations The supraorbital foramen/notch—SOF; The infraorbital foramen—IOF; The mental foramen—MFO; SD-standard deviation, *p* < 0.05 significant value.

**Table 3:** Predictive values of FA1 and FA2 indices for distinct measurements with selected cutpoints. PPV—Positive Predictive Value, NPV—Negative Predictive Value, AUC—Area Under [ROC] Curve.

Predictor	Cutpoint	Sensitivity (%)	Specificity (%)	PPV (%)	NPV (%)	Youden’s Index	AUC
IOF-MFO FA1	0.510	70%	90%	77.78%	85.71%	0.600	0.838
IOF-MFO FA2	0.078	70%	95%	87.50%	86.36%	0.650	0.865
SOF-MFO FA1	0.410	90%	80%	69.23%	94.12%	0.700	0.912
SOF-MFO FA2	0.039	90%	80%	69.23%	94.12%	0.700	0.925
SOF-IOF FA2	0.0315	70%	80%	63.64%	84.21%	0.500	0.770
SOF-IOF FA1	0.15	70%	80%	63.64%	84.21%	0.500	0.753
Total FA2	0.129	90%	90%	81.82%	94.74%	0.800	0.940

Abbreviations The supraorbital foramen/notch—SOF; The infraorbital foramen—IOF; The mental foramen—MFO; SD-standard deviation, *p* < 0.05 significant value.

## Data Availability

Availability of supporting data. The datasets used and/or analyzed during the current study are available from the corresponding author on reasonable request.
